# Mannose 6-Phosphate Receptor Is Reduced in -Synuclein Overexpressing Models of Parkinsons Disease

**DOI:** 10.1371/journal.pone.0160501

**Published:** 2016-08-10

**Authors:** Carmela Matrone, Nicolas Dzamko, Peder Madsen, Mette Nyegaard, Regina Pohlmann, Rikke V. Søndergaard, Louise B. Lassen, Thomas L. Andresen, Glenda M. Halliday, Poul Henning Jensen, Morten S. Nielsen

**Affiliations:** 1 Department of Biomedicine, Faculty of Health, Aarhus University, 8000 Aarhus C, Denmark; 2 Neuroscience Research Australia, Sydney, NSW 2031, and School of Medical Sciences, Faculty of Medicine, The University of New South Wales, Sydney, NSW 2052, Australia; 3 Research Initiative on Blood Brain and Drug Delivery, The Lundbeck Foundation, 8000 Aarhus C, Denmark; 4 Institute of Physiological Chemistry and Pathobiochemistry, University of Münster, 48149 Münster, Germany; 5 Department of Micro- and Nanotechnology, Technical University of Denmark (DTU) Nanotech, DTU, 2800 Lyngby, Denmark; Louisiana State University Health Sciences Center, UNITED STATES

## Abstract

Increasing evidence points to defects in autophagy as a common denominator in most neurodegenerative conditions. Progressive functional decline in the autophagy-lysosomal pathway (ALP) occurs with age, and the consequent impairment in protein processing capacity has been associated with a higher risk of neurodegeneration. Defects in cathepsin D (CD) processing and α-synuclein degradation causing its accumulation in lysosomes are particularly relevant for the development of Parkinson's disease (PD). However, the mechanism by which alterations in CD maturation and α-synuclein degradation leads to autophagy defects in PD neurons is still uncertain. Here we demonstrate that MPR300 shuttling between endosomes and the trans Golgi network is altered in α-synuclein overexpressing neurons. Consequently, CD is not correctly trafficked to lysosomes and cannot be processed to generate its mature active form, leading to a reduced CD-mediated α-synuclein degradation and α-synuclein accumulation in neurons. MPR300 is downregulated in brain from α-synuclein overexpressing animal models and in PD patients with early diagnosis. These data indicate MPR300 as crucial player in the autophagy-lysosomal dysfunctions reported in PD and pinpoint MRP300 as a potential biomarker for PD.

## Introduction

Emerging evidence points to an imbalance between synthesis and degradation of proteins as a leading cause for the aggregation of redundant proteins into insoluble deposits that impair the functions of neurons and contribute to the onset and progression of neurodegenerative disease, such as Parkinson's (PD) and Alzheimer’s disease [1, 2]. PD is the most common neurodegenerative movement disorder characterized by the severe degeneration of neurons in the substantia nigra pars compacta and progressive changes in other brain regions [3–5]. At the cellular level, neuronal loss is preceded by synapse degeneration and the presence of cytoplasmic inclusions known as Lewy bodies, largely consisting of fibrils formed from insoluble α-synuclein (α-syn) protein [4, 6, 7].

α-Syn is a synaptic protein mainly localized in the proximity of synaptic vesicles [8, 9]. The various physiological roles of α-syn are still under investigation, however it is known to bind lipid membranes on synaptic vesicles, mitochondria and the Golgi apparatus [10]. Accordingly, α-syn serves as a molecular chaperone, facilitating SNARE complex assembly at the synapse [11, 12]. Furthermore, it controls the dynamics of neurotransmitter release and clathrin-dependent replacement of the pool of synaptic vesicles [13], as well as participating in the stabilization of mitochondrial membrane proteins [14]. The intracellular homeostasis of α-syn requires the proper degradation of the protein by three mechanisms: chaperone-mediated autophagy, macroautophagy and the ubiquitin-proteasome system. Impairment of these pathways is hypothesized to be an important pathogenic factor in sporadic forms of PD [15, 16] whose relative contributions can be subjected to several regulatory mechanisms, e.g. by aging [17]. The impaired cell trafficking in the autophagy-lysosomal pathway (ALP) results in the exosomal release of α-syn [1] and likely of other target proteins.

Cathepsin D (CD) is a lysosomal protease previously demonstrated to cleave α-syn and decrease its toxicity in cell lines [18]. Additionally, overexpression of CD reduces α-syn aggregation in cell models and CD deficiency increases the neuronal aggregation of α-syn [19, 20]. CD is synthesized as an inactive pro-form (proCD) in the endoplasmatic reticulum. Upon final maturation of proCD in the trans Golgi-network (TGN) and the glycosylation with mannose 6-phosphate, the protein is translocated to endosomal compartments. The transport of CD from the TGN to endosomes is mediated by the cation-independent mannose-6-phosphate receptor, a type-1 receptor also known as the 300 kDa mannose-6-phosphate receptor (MPR300). In the endosomes, proCD is released from the receptor and retained in the compartment during its maturation and endosomal trafficking into lysosomes. MPR300 recycles back to the TGN to initiate a new cycle [21].

Here we investigated whether alterations or defects in proteins involved in α-syn trafficking might be involved in PD and thus be exploitable as prognostic indicators. We found CD levels are significantly reduced in brain tissue from transgenic (homozygote and heterozygote) α-syn-overexpressing mice models of PD. The reduction in CD results from defective trafficking and sorting. This trafficking deficit is related to a decrease in MPR300 levels in neurons, with a large amount of MPR300 being processed into lysosomes and/or released into the media of α-syn overexpressing cells and neurons, or found in the soluble protein brain tissue fraction of α-syn overexpressing mice. Furthermore, MPR300 levels were also decreased in human brain tissues from patients in the early stages of PD compared to aged matched healthy controls. All together these results point to a new unsuspected player in controlling α-syn degradation in neurons. MPR300 reduces proCD transport to late endosomes and thereby the quantities of mature CD in lysosomes. The resultant defect in CD activity blocks α-syn degradation leading to its intracellular accumulation and to lysosomal dysfunction. The crucial increased release of MPR300 and its concomitant decrease in intracellular levels, also supported by data from PD patients, point to MPR300 as a valuable potential tool in PD.

## Materials and Methods

### Human tissue samples and tissue processing

Human tissue samples from the anterior cingulate cortex of autopsy-confirmed sporadic PD subjects (n = 6, 4 men and 2 women, age 76±2) and matched clinical and neuropathological controls (n = 9, 5 men and 4 women, age 74±3) were provided by the Sydney Brain Bank following appropriate institutional approvals. In order to assess early PD, cases were restricted to α-syn pathology at Braak stage 4. At this stage the anterior cingulate cortex has increased SDS-soluble α-syn, but does not yet have Lewy body pathology [22]. All PD cases met the UK Brain Bank clinical criteria for a diagnosis of PD and had no other neurodegenerative conditions. The cases analysed have been previously used to assess lipids, and α-syn levels in early PD [22].

As previously described [22], Tris-buffered saline (TBS) and SDS-soluble proteins were serially extracted from 250 mg of fresh-frozen brain tissue from each case. Briefly, tissue was mechanically homogenized in 10 volumes TBS homogenization buffer (50 mM Tris, 125 mM NaCl, pH 7.4, 5 mM EDTA, 0.02% sodium azide) containing protease inhibitor cocktail (Roche), followed by centrifugation at 120,000 x g for 2 h at 4°C, with the supernatant collected as the TBS-soluble fraction containing cytosolic proteins. The pellet was resuspended in SDS solubilization buffer (TBS homogenization buffer containing 5% SDS), sonicated (2 x 10 s bursts) and centrifuged at 100,000 x g for 30 min at 25°C, with the supernatant collected as the SDS-soluble fraction containing membrane-associated proteins. Protein concentrations were measured using a bicinchoninic acid assay (Pierce BCA Protein Assay Kit, Thermo Scientific), following the manufacturer’s instructions with samples then stored at -80°C until use.

### Animal strains

The animals used for these studies were backcrossed to C57Bl/6J mice. The mice were bred and group housed in the Lab Animal Centre of Taconic (Aarhus, DK) at an ambient temperature of 22–23°C and on a 12/12 h dark/light cycle (lights on 7 a.m.). A total of fourteen 3-month, four 6-month and four 12-month old WT and ASO^Tg/Tg^ male, 3, 6 and 12-month-old ASO^Tg/+^ and four 3-month old WT and 6 ASO^Tg/Tg^ male mice were used for experiments. Primary cultures were obtained from 6 WT and 6 ASO^Tg/Tg^ pregnant mice. Prior to euthanasia, all of the mice were anesthetized by 2-bromo-2-chloro-1,1,1-trifluoroethane inhalation and euthanized in accordance with international guidelines on the ethical use of animals (European Communities Council Directive of November 24, 1986; 86/609/EEC) and Danish guidelines. Heterozygous transgenic mice with mixed C57BL/6-DBA/2 background and expressing human α-syn under the Thy-1 promotor were a kind gift from Dr. Eliezer Masliah and have previously been described in detail [23]. Homozygous α-syn mice (backcrossed in C57BI6 for at least 9 generations), overexpressing wild-type human α-syn under the control of the partial mouse α-syn promoter, were provided by Dr P.H. Kallunki from A.S. Lundbeck and have previously been described in detail [24, 25].

### Cell cultures and neuronal primary cultures

SH-SY5Y cells were transfected using Fugene6 Transfection Reagent (Promega) with an α-syn construct in pCDNA3.1(-)(Zeo) (Invitrogene). 48 h after transfection, cells were transferred to 96 wells plates in culture medium supplemented with Zeocine. Stable clones were tested for α-syn expression using western blot (WB) and a single clone with homogeneous α-syn staining selected from the positive clones (from hereon referred to as ASO cells).

Cortical neurons were prepared from embryonic day 17/18 (E17/E18) mice, as previously reported [26]. Neurons were dissected in HBSS buffered with HEPES and dissociated via papain treatment. A total of 200,000 cells were plated on slides pre-coated with poly-l-lysine. After 2 d of culturing in neurobasal medium with B-27 supplement and glutamax, cytosine arabinofuranoside was added to reduce glial proliferation. ASO hippocampal neurons appeared very difficult to keep in culture when compared to the correspondent background matched WT, starting to dye after 10 days of culturing and showing an intense glial cell proliferation. To avoid these difficulties, immunofluorescence and WB analysis were performed on 10 day plated hippocampal neurons.

NH_4_Cl and chloroquine were obtained from Sigma Chemical (DK). 5M NH_4_Cl and 50mM chloroquine stock solutions were made in sterile distilled water. Each drug was used at the lowest concentration needed to have an effect (NH_4_Cl 5mM; Chloroquine 10μM), and neurons were treated for the minimum amount of time required to see an effect on MPR300 vesicle number (6 h) to minimize secondary effects of any of these drugs [27]. Cell viability was assessed before and after drug exposure both in CTRL and ASO cells.

### Western blotting

Equal amounts (30 μg) of soluble animal and cellular proteins and SDS-soluble human tissue proteins were separated on 4–12% Bis-Tris SDS-PAGE gels (Invitrogen, DK or Novex system, Life Technologies), blotted onto nitrocellulose membranes (Amersham, DK or Biorad), and incubated overnight with the appropriate primary antibody (see below). Visualisation of protein bands was performed on a Chemidoc MP imaging system (Biorad) and Image Lab software (Biorad) used to quantify relative protein intensity with β-actin used to normalise for protein loading.

Media from control and ASO cells were processed following the procedure previously described by Matrone *et al*. [28]. Shortly 1.500.000 control and ASO cells were plated in 2ml of media. After 24hrs media was collected and ultra-centrifuged at 100000rpm for 12hrs. Cells were processed for WB analysis. Pellets obtained after ultracentrifugation were suspended in 100ul of loading buffer containing SDS 2% and 1/10 of their total volumes were loaded on the gel for WB analysis. Hsc70 protein was used as protein loading control for quantification of relative protein intensity [29].

The antibodies for WB and immunofluorescence were as follow: rabbit-anti-α-syn (ASY1) [30, 31], mouse-anti-EEA1 (Abcam), mouse-anti-α-syn (BD biosciences), rabbit-anti-human MPR300 (2C2) [32], goat-anti-MPR300 (clone k21, Santa Cruz Biotech), anti-MPR300 (Abcam), goat-anti-Cathepsin D (Santa Cruz Biotech), sheep-anti-TGN46 (AbD Serotec), rabbit-anti-Lamp1 (clone ab24170, Abcam), rabbit-anti-Rab4 (BD Transduction), rabbit-anti-Rab7 (Abcam), rabbit-anti-GFP (Sigma), rabbit-anti- β -actin (Sigma), rabbit anti-β-actin (Abcam) and Alexa-fluor labelled secondary antibodies (Invitrogen).

### Animal brain tissue and cellular fractionations

Whole mouse brains were sonicated in a lysis buffer containing 20 mM Tris-base pH 7.4, 250 mM sucrose, 1 mM EDTA, 1 mM EGTA plus protease (Roche, Complete) and phosphatase inhibitors. For soluble vs. insoluble fractionations, 250μg hippocampal tissues from each strain were homogenized in 300μl of lysis buffer and the lysates were firstly spun at 1,000 g for 15 min and then for an additional 4 h at 100,000 g. The soluble fraction was separated from the pellet and analysed by WB loading 1/10 of the total volume (30μl). Pellets were suspended in 100μl of loading buffer containing β-mercaptoethanol and 10μl of each sample was loaded and analysed by WB.

### Vesicular pH assay

Lysosomal pH was determined in SH-SY5Y cells and stably transfected α-syn SH-SY5Y cells (ASO cells) as previously described [33]. Cells were incubated in the presence of 10 μg/mL nanoparticle for 24 h at 37°C in normal growth medium. The cells were then washed three times with ice-cold heparin (20 units/mL in PBS), once with PBS and kept in growth medium without phenol red for observation by confocal microscopy. Cells were either imaged immediately or treated with 100 nM Bafolimycin A_1_ for 45 min before imaging. Images were captured with a Leica TCS SP5 confocal microscope with a 63x water/immersion objective (Leica Microsystems, Germany). The microscope was equipped with an incubator box and CO_2_ supply for optimal growth conditions during imaging (Life Imaging Services, Switzerland). Images were acquired with fixed settings for all samples and the corresponding calibration curve. Two-color images were obtained by sequential line scanning with the following excitation/ emission wavelengths: 488/493-560 nm and 561/566-680 nm. The calibration curve was prepared by diluting the nanosensor in buffers (20 mM phosphate/20 mM citrate/20 mM malate/100 mM NaCl) from pH 2.8–7.5 with a final nanosensor concentration of 8 mg/mL. 2.5 μL of each calibration solution was transferred to a diagnostic microscopy slide, sealed with a cover glass and imaged. Image analysis was performed as described previously with a pixel based method [34]. Briefly, image processing was used in order to determine which pixels are actual signal from nanosensors and the included pixels were then converted to pH via the calibration curve. pH histograms are presented as mean ± SEM. 800–1000 data points were analysed for each cell line.

### Confocal microscopy and high content screening analysis

Cells and neurons were fixed for 20 min in PBS containing 4% formaldehyde, permeabilized with 0.25% Saponin (5–10 min, 20°C), and processed for single labelling with the appropriate antibody. Secondary antibodies coupled to Alexa dyes (488 or 594) were from Invitrogen. Nuclei were visualized by staining with Hoechst dye 33258 (1 μg/ml) (Sigma). Digital images were obtained with a Zeiss LSM confocal lsm780 system using 63× oil NA 1.3 objective. Quantification of the co-localization experiments was performed using Zen 2009 software. Pearson coefficients (R coefficients) were used as co-localization coefficients, following the same procedure previously described by La Rosa et al., 2015 [35]

Olympus automated Scan^R imaging High Content imaging station base on Olympus BX73 microscope, were used for quantitative analysis [36, 37]. Images were acquired with a 40 times objective, single band emission filters for Hoechst 33258, Alexa-Fluor-488 and Alexa-Fluor-568, and a Hamamatsu EMMCD camera. Acquired single-layer images were background-subtracted before image analysis, after which an edge-detection algorithm was used for segmentation of nuclei and stained vesicles. This algorithm use gradient intensities in the image to identify vesicles. If a closed connecting line (edge) can be drawn around an object, the object is segmented, and the selection is independent of the vesicle shapes. However, vesicles larger than 3 μm in diameter were not marked. Images with artifacts or zero cells were gated out, where after the number of vesicles per cell was determined by calculating for each image the ratio of vesicles to nuclei. The number, area, and morphology of vesicles from at least 10^4^ cells for each experiment (5 experiments in triplicate) were detected and analysed.

### Real-time PCR for mRNA quantification

Total RNA was isolated from cortex of transgenic mice overexpressing α-syn (n = 6) and non-transgenic littermates (n = 4) using NucleoSpin RNA kit (Macerey-Nagel). The cDNA was synthesized from 0.5 μg total RNA in 20 μl total reaction volume using an iScript cDNA synthesis kit (Biorad). The primer sequence is shown below. Real-time PCR was performed with a Lightcycler 480 SYBR Green I master mix on a LightCycle480 instrument (Roche). Primers for the two mouse normalizer genes (*Ywhaz* and *Gapdh*) were purchased from PrimerDesign Ltd (Rownhams, UK). Intron spanning primer pairs for *Igf2r* and *Ctsd*, were designed using Primer3 [38]. Standard curves were performed for all genes to ensure efficiency above 90%. Minus RT and blank water controls were included. Normalization was performed using the average of *Ywhaz* and *Gapdh*. Fold change for all samples was calculated by comparing to the average of the four Wt mice. To compare the RNA levels between the two groups (transgenic mice overexpressing α-syn and WT mice), all fold changes were log transformed to ensure normal distribution and a two-sided unpaired t-test performed.

### Statistical analysis

Data were expressed as means ± SEM. The various statistical tests used are indicated in the figure legends. We performed statistical analysis using GraphPad Prism (version 5.0c, USA). In experiments involving two experimental groups and a single, non-repeated, dependent variable, data were analysed using Student's t-test. When experiments involved three groups of animals or repeated measurements, the means were compared using, as required, either one- or two-way analysis of variance (ANOVA). Post hoc comparisons were made using Tukey's test, when appropriate.

Multivariate analysis using SPSS (IBM, Chicago, IL), covarying for age and post-mortem delay, was used to determine significant differences (set at p < 0.05) in MPR300 or α-syn protein levels in human brain tissue.

## Results and Discussion

### α-Synuclein downregulates Cathepsin D

**Fig 1:**
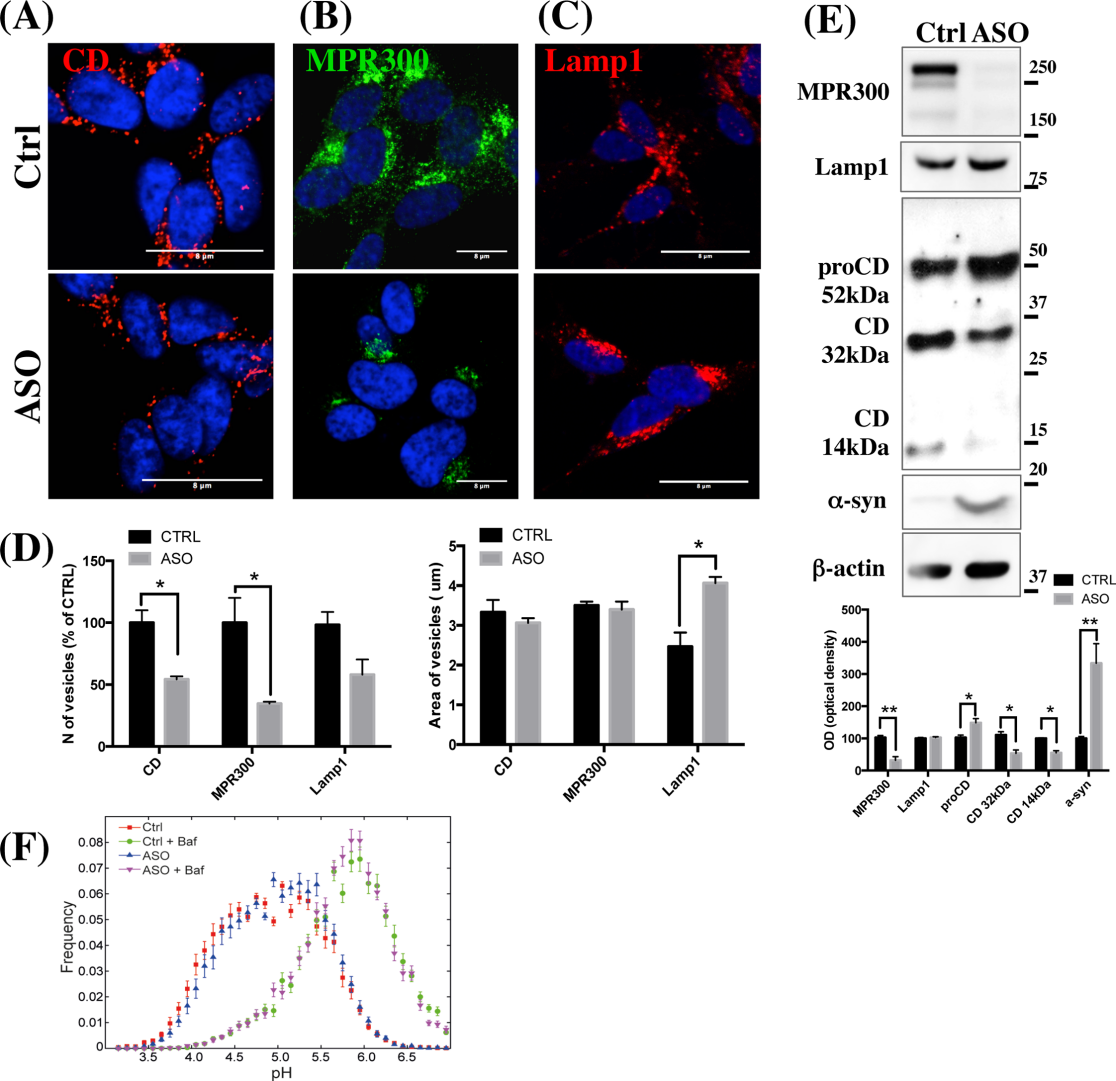
CD and MPR are reduced in α-syn expressing SH-SY5Y cells. (A, B, C) Confocal microscopy analysis of single immunostaining for CD, MPR300 and Lamp1, respectively, in Ctrl and α-syn expressing SH-SY5Y cells (ASO). Scale bar 8 μm. Panels are representative of 3 different experiments. Images were also acquired on an automated Olympus microscope and the number and the area (D) of the vesicles pr. nuclei quantified using Scan^R imaging software (Olympus) (three slides per condition). (N = 5; Two tailed T-test) *P<0.05 vs Ctrl. The total number of vesicles was normalized to DAPI positive cells and expressed as media of vesicles per cell. (E) WB analysis for proCD and CD cleaved peptides migrating at 32kDa and 14kDa, MPR300, Lamp1 and α-syn from total lysate (30μg) of human stable-transfected α-syn overexpressing cells (ASO) and their correspondent empty-vector control (Ctrl) cells. A representative picture showing the levels of α-syn in each sample is shown below. The optical density analysis is reported below. Data were normalized on the basis of the correspondent β-actin values and expressed as % of Ctrl. The experiments were run 3 times in triplicate. (N = 3; Two tailed-T Test). (*) p<0.05 and (**) p<0.01 vs Ctrl. (F) Ctrl and stable-transfected α-syn SH-SY5Y cells were treated with triple-labelled pH-sensitive nanosensors for 24 h and imaged by confocal microscopy. The ratio of the intensity signals from the pH-sensitive and reference fluorophores was converted to pH via the calibration curve. To ensure the assay was working, an increase in vesicular pH was induced in Ctrl and ASO cells by treatment with 100 nM Bafilomycin A_1_ that blocks the vacuolar-type H^+^-ATPase. Mean ± SEM (number of slides n = 10) are presented. A maximum of 7% and 0.5% of measurements fell outside the range of the nanosensor for the bafilomycin A_1_-treated and untreated samples, respectively. Each experiment was repeated three times with similar results and for each experiment between 800 and 1000 data points were acquired for every cell line.

A large body of data points to CD as a modulator of α-syn degradation in lysosomes [18–20, 39, 40], however the interplay between CD and α-syn pathway in PD is still unclear. In order to analyse how α-syn overexpression compromises lysosomal and CD activity, we firstly performed confocal microscopy analysis for CD in a α-syn overexpressing SH-SY5Y cell line (ASO cells) (Fig 1A) and assessed the number of CD positive vesicles pr. nuclei relative to controls (Ctrl) (Fig 1D). We found that the number of CD-positive vesicles from ASO cells was around half that of controls, while the area of the vesicles did not change (Fig 1D). Alterations in the CD pathway can be indicative of a lysosomal deficit [41]. Consistent with this, confocal microscopy analysis indicated the presence of dense Lamp1-positive areas in ASO cells, suggesting that lysosome vesicles were likely fused to form bigger condensed areas (Fig 1C). Notably, while the area of Lamp1-positive vesicles was increased, the number of Lamp1-positive vesicles was not significantly changed (Fig 1D), which is a typical phenotype for cells with reduced lysosomal activity [41]. Consistently, WB analysis did not show significant differences in the Lamp1 expression levels between Ctrl and ASO cell line (Fig 1E). Additionally, WB analysis revealed a reduction in the mature form of CD (32 kDa and 14 kDa) and an increase in proCD levels in ASO cells (Fig 1E). Alteration in the pH in vesicles is considered to play an important role in aging and neuronal degeneration [42] and the vesicular pH in lysosomes is responsible for the maturation of proCD to active CD [43]. It has been previously demonstrated that the A53T α-syn mutation affects lysosomal acidification in PC12 cells, which leads to lysosomal dysfunction [44]. In order to evaluate whether the reduction in CD cleavage was due to alteration in the pH in the lumen of the lysosomes, Ctrl and ASO cells were incubated for 24 h with triple-labelled pH sensitive nanosensor [45]. As shown in Fig 1F, vesicular pH in the lysosomal vesicles from ASO cells (blue curve) was not significantly different to that of Ctrl cells (SH-SY5Y, red curve) demonstrating that the defect in proCD conversion to CD was not ascribable to alterations in lysosomal pH. As a positive control for these experiments, ASO and Ctrl cells were incubated with 100 nM Bafilomycin A1 (purple curve and green curve, respectively). Bafilomycin increases vesicular pH by blocking the vacuolar-type H^+^-ATPase, but does not interfere with nanosensor [33, 34]. As indicated by the purple and green curves, both ASO and Ctrl pH values increased under Bafilomycin exposure, denoting that the experiments were running correctly.

### MPR300 expression is reduced in α-syn overexpressing (ASO) cell lines and in ASO mice

Since the decreased CD processing in ASO cells was not due to alteration of vesicular pH, and since the processing of CD is largely related to its MPR300-mediated trafficking towards endosomes and lysosomes [32], we hypothesized that MPR300 expression, trafficking and sorting might be affected in our experimental conditions. First we assessed MPR300 levels in ASO and Ctrl cells by IF and WB (Fig 1B and 1E) and found that MPR300 expression levels were indeed decreased in ASO cells. In addition, as for CD, we noted a clear decrease in the number of MPR300-positive vesicles in ASO cells (Fig 1D) that was consistent with the detected decrease in protein expression levels. Interestingly, a correlation between an increased area and density of lysosomes and reduced MPR300-mediated transport of CD and other acidic hydrolases has previously been described under different experimental conditions [32, 46].

**Fig 2:**
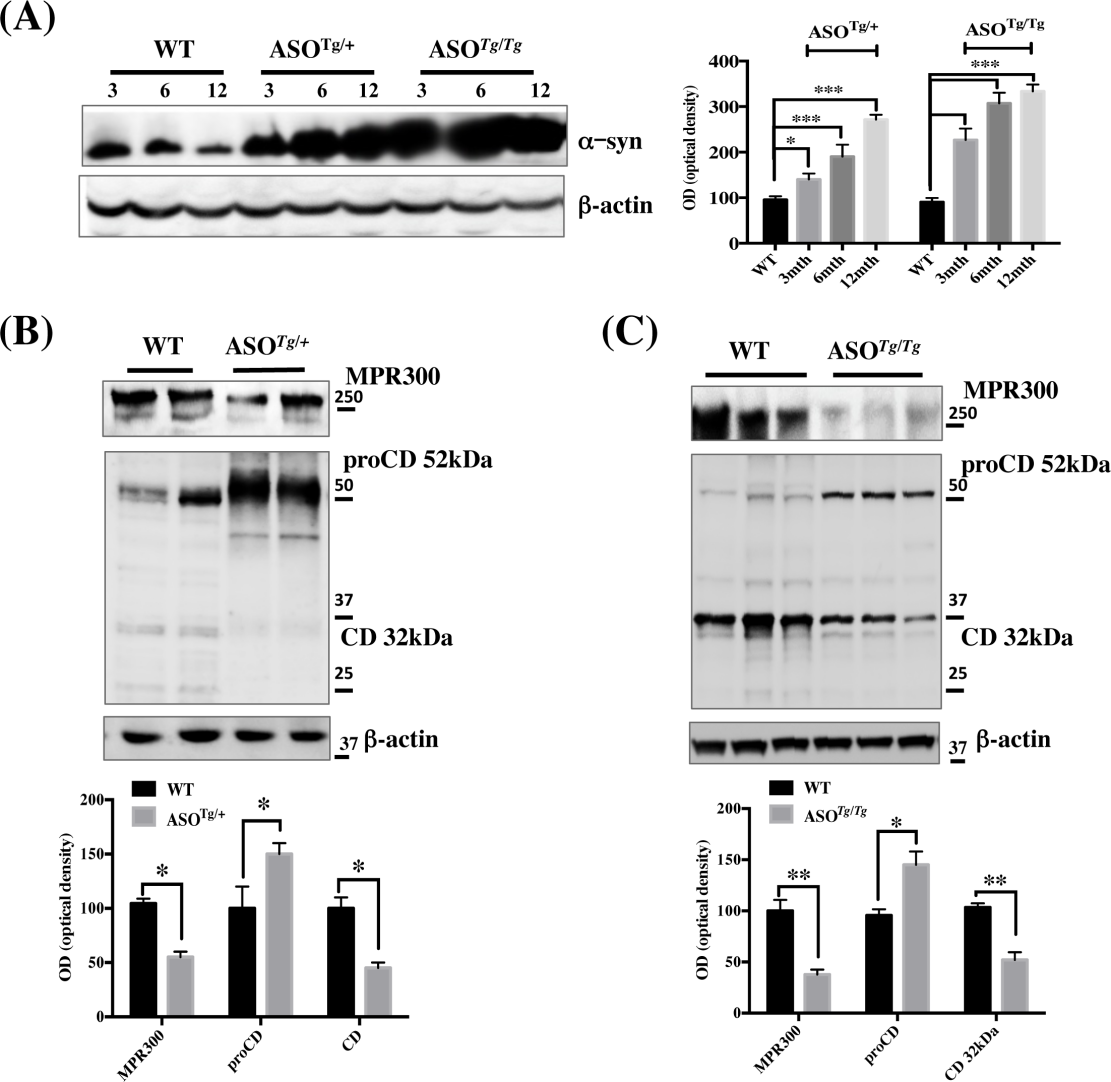
CD and MPR300 protein levels are reduced in α-syn overexpressing mice cortex. (A) WB analysis for α-syn performed in total lysate from cerebral cortical tissues of 3, 6 and 12 month-aged WT, ASO^*Tg/+*^ and ASO^*TgTtg*^ mice. WT tissues are from C57BL/6J mice (background matched to ASO^*Tg/Tg*^*)*. No obvious differences in α-syn expression were detected between the two WT mice with different background. N = 5; One-way Anova with post-hoc Tukey test. (B, C) WB for MPR300 and proCD/CD in total lysate from cortex of heterozygous α-syn ASO^*Tg/+*^ and homozygous α-syn ASO^*Tg/Tg*^ mice respectively and their correspondent background matched controls. Charts of the band optical density (OD) are shown below each panel. Data were normalized to the corresponding β-actin values and expressed as % of WT. (*) p<0.05 vs WT. The experiments were run 5 times in triplicate. N = 5. Two tailed T-Test.

To further analyse the mechanism by which α-syn overexpression affects MPR300 expression we employed two different strains of α-syn-overexpressing mice (heterozygote, ASO^*Tg/+*^, and homozygote, ASO^*Tg/Tg*^) [23–25]. We isolated cortex from 3, 6 and 12 month-old mice and assessed α-syn protein levels by WB (Fig 2A). We noted that in both ASO^*Tg/+*^ and ASO^*Tg/Tg*^ mice a significant increase in α-syn levels was already detectable at 3-months, and this level increased further with age (Fig 2A). Notably, we did not detect any obvious difference in α-syn levels between the WT control samples with different background (Fig 2A, chart). Since we were interested in the early events occurring in ASO^*Tg/+*^ and ASO^*Tg/Tg*^ mice, we decided to focus all our studies on 3-month-old mice. Consistent with our findings in ASO cell lines, MPR300 levels and the mature CD, migrating at 32 kDa, were significantly reduced, while proCD was higher in both ASO^*Tg/+*^ and ASO^*Tg/Tg*^ cortical tissues (Fig 2B and 2C). In contrast, the mature 14 kDa band of CD was only weakly visible in WT samples and almost completely undetectable in ASO^*Tg/+*^ and ASO^*Tg/Tg*^ tissues (data not shown). Notably, the mRNA levels of both CD and MPR300 were not different between WT and ASO mice (Table 1). Overall these data support our hypothesis that defects in MPR300 receptor likely play a role in the reduction of active CD in lysosomes and consequently in the lysosomal deficiencies observed in ASO cells.

**Table 1:** qPCR on mRNA from ASO^*tg/tg*^ and wildtype mice brain tissues. The tested genes encode CD (ctds), MPR300 (Igf2r), SorLA (Sorl1), Sortilin (sort1), AP-1μ chain (Ap1m1) and AP-1γ chain (Ap1g1). For each gene, all ASO^*tg/tg*^ samples were normalised to the average of the 4 wildtype sample. The values in parenthesis are the standard deviation on the fold change within each group.

Gene ID	Aso (n = 6)	Wt (n = 4)	p-value
**Ctds**	1.01 (0.09)	1.00 (0.07)	0.87
**Igf2r**	1.03 (0.11)	1.00 (0.04)	0.82
**Sorl1**	0.95 (0.19)	1.00 (0.07)	0.58
**Sort1**	1.01 (0.24)	1.00 (0.16)	0.98
**Ap1m1**	0.96 (0.10)	1.00 (0.10)	0.56
**Ap1g1**	1.04 (0.14)	1.00 (0.10)	0.68

### A rearrangement in MPR300 trafficking affects CD cleavage in ASO mice

**Fig 3:**
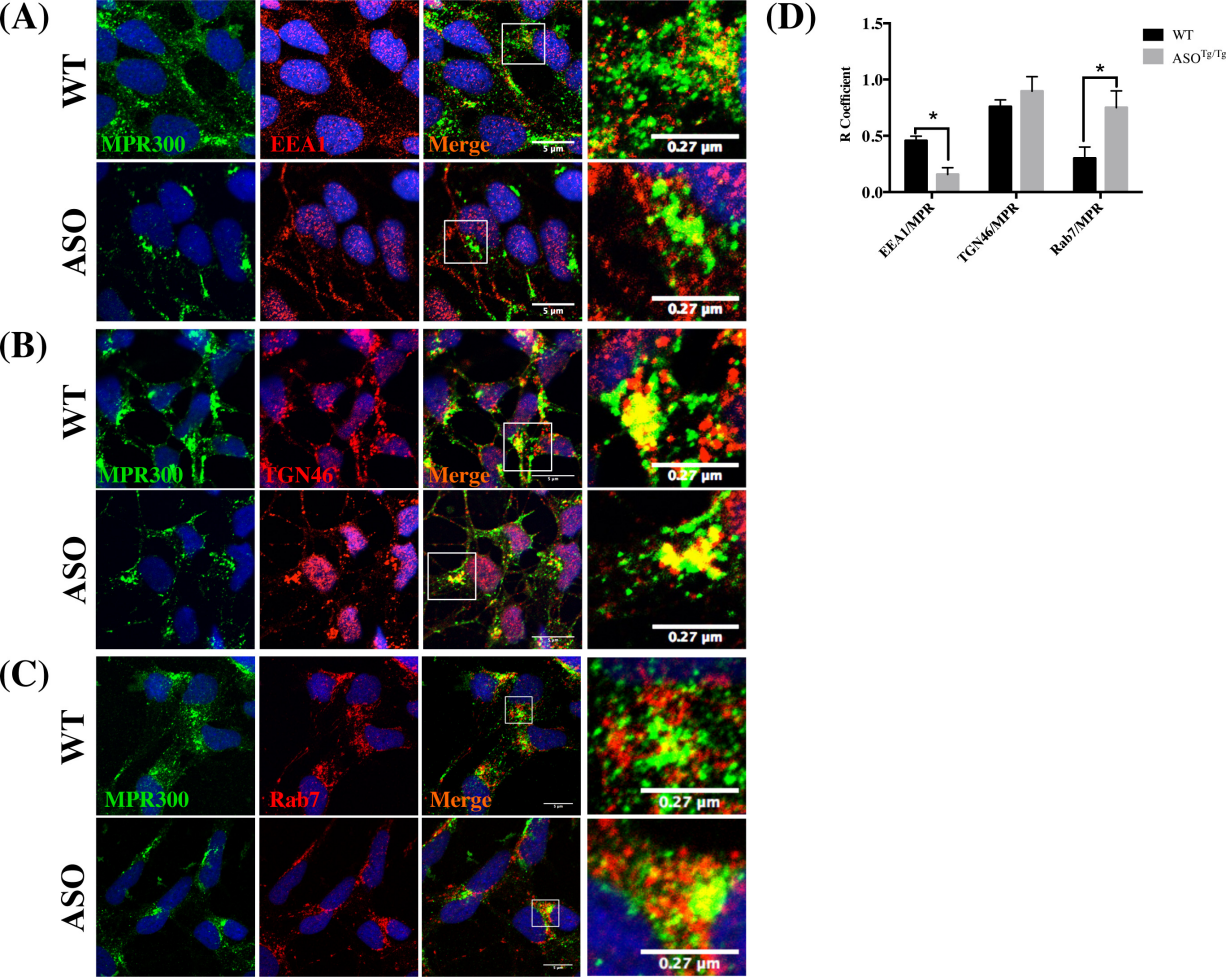
MPR300 preferentially localizes in late endosomes in ASOTg/Tg neurons. (A-C) Confocal microscopy analysis of MPR300 (green) and EEA1, TGN46 and Rab7 (red), respectively, in WT and ASO^*Tg/Tg*^ cortical neurons. Scale bar 5 μm. (D) The extent of MPR300 co-localization to EEA1, TGN46 and Rab7 is reported in panel D. Quantitative analysis was performed using Zen software. The (R) coefficient (Pearson’s coefficient) was used for the quantitative and comparative analyses (R). Data are expressed as mean ± SEM. n = 8. *p<0.05. Two tailed T-Test).

To dissect the mechanism leading to the reduction of MPR300 in ASO^*Tg/Tg*^ tissues, we firstly questioned whether MPR300 trafficking was altered. Since MPR300 is shuttled between the TGN and endosome and only a small amount reaches the lysosome under normal conditions [47, 48], we analysed its localization in the early (EEA1), trans Golgi network (TGN) and late endosome (Rab7) (Fig 3A–3C). We hypothesized that the reduction of MPR300 from ASO^*Tg/Tg*^ neurons might be due to its inefficient retrograde transport from the endosomes to TGN. Such a defect would likely be followed by an augmented MPR300 transport to the late endosome with its consequent degradation in the lysosome or secretion. Therefore, we assessed whether MPR300 was increased in late endosomes (Rab7-positive vesicles). MPR300 co-localization analysis performed with anti-Rab7 antibody clearly identified an increase in MPR300 in Rab7-positive vesicles from ASO^*Tg/Tg*^ neurons as the area of overlapping Rab7 and MPR300 increased (Fig 3C and 3D). In contrast MPR300 was reduced in the EEA1-positive vesicles from ASO^*Tg/Tg*^ neurons (Fig 3A and 3D). Differently, we didn’t assess significant difference in the extent of co-localization between MPR300 and TGN46 positive vesicles (Fig 3B and 3D).

**Fig 4:**
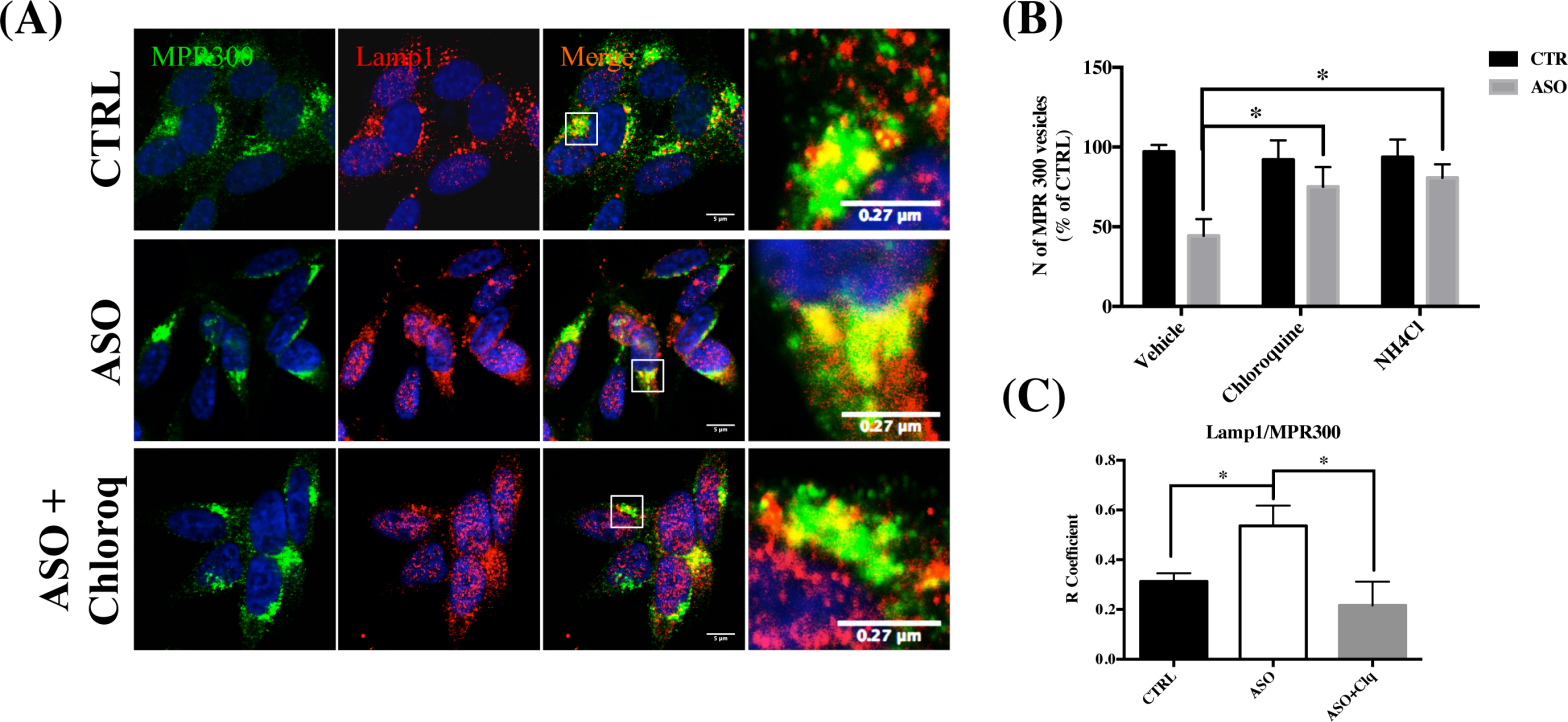
The reduction in MPR300 levels is partially controlled by the exposure to lysosome inhibitors. (A) Confocal microscopy analysis of MPR300 (green) and Lamp1 (red) positive vesicles. To counteract the decrease of MPR300 vesicles, Ctrl and ASO cells were exposed to vehicle, Chloroquine (10mM) and NH_4_Cl (5mM) for 6 hrs. Images were also acquired on an automated Olympus microscope and the number of the vesicles quantified using Scan^R imaging software (Olympus) (three slides per condition) (B). (N = 3; One-way Anova with post-hoc Tukey test) *P<0.05 vs Ctrl. The extent of MPR300 co-localization to Lamp1 positive vesicles is reported in panel C. Quantitative analysis was performed using Zen software. The (R) coefficient (Pearson’s coefficient) was used for the quantitative and comparative analyses (R). Data are expressed as mean ± SEM. n = 4. *p<0.05 One-way Anova with post-hoc Tukey test).

We hypothesized that the reduction in MPR300 levels could be related to its altered trafficking toward late endosomes and possibly also to lysosomes and we questioned whether lysosome inhibitors might rescue the MPR300 vesicles defect. To this aim we either inhibited trafficking to lysosomes directly (chloroquine) or inactivated acidic hydrolases (NH_4_Cl) and we noted that the number of MPR300 positive vesicles was significantly increased in response to both chloroquine and NH_4_Cl inhibitors exposure in ASO cells (Fig 4B). Notably, the extent of co-localization between MPR300 (green) and Lamp1 (red) immunofluorescence appeared to be reduced in presence of chloroquine, likely suggesting a partial recovering of MPR300 trafficking and sorting under lysosomal inhibitor exposure (Fig 4A and 4C).

**Fig 5:**
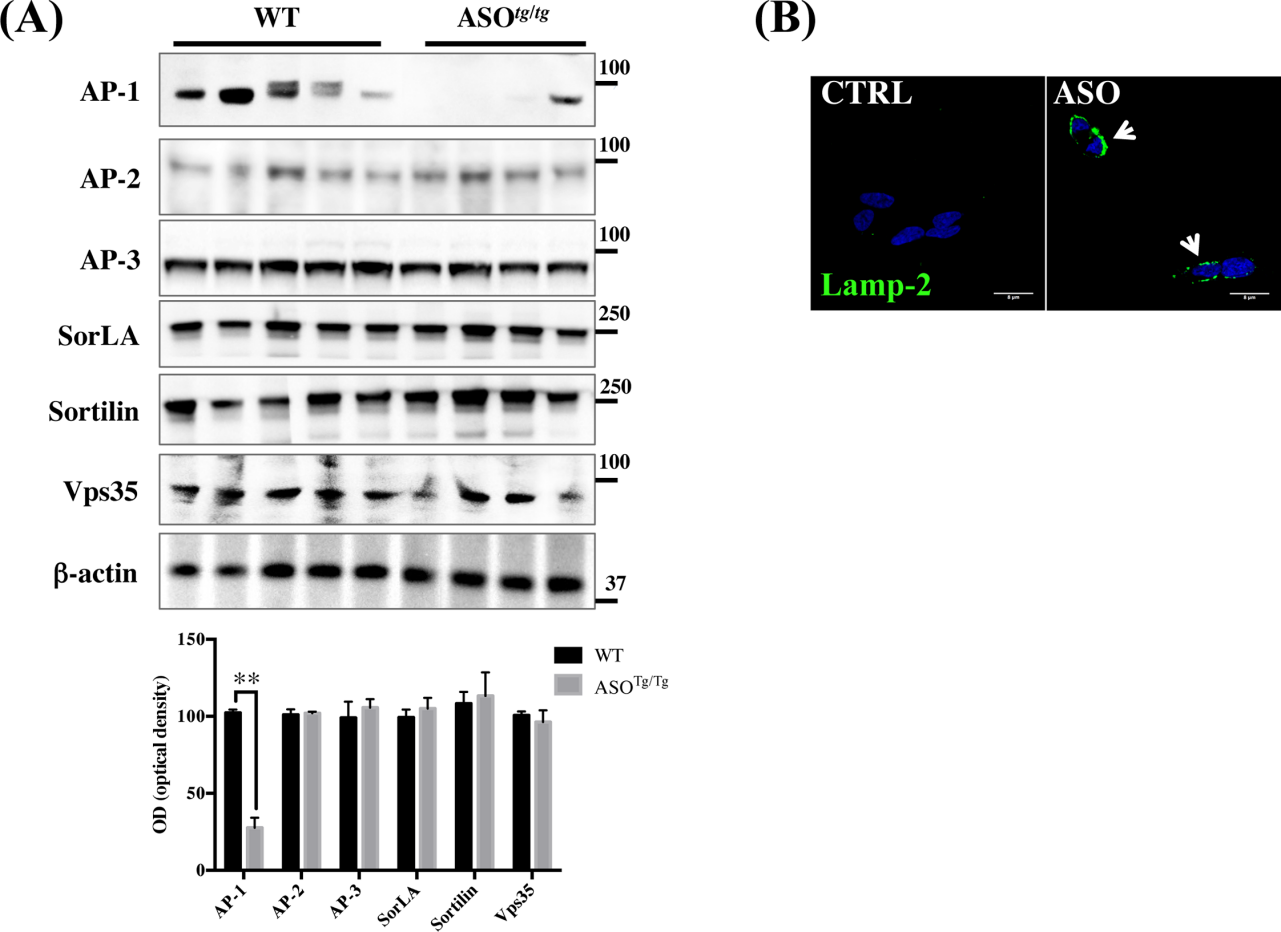
AP1 protein levels are reduced in α-syn overexpressing mice cortex. (A) Western blot analysis of AP-1, AP-2, AP-3, SorLA, Sortilin and VPS35 from WT and ASO^*Tg/Tg*^ cerebral cortical tissues. Optical density analysis is reported below. Data were normalized on the basis of the correspondent β–actin values and expressed as % of WT. The experiments have been run 5 times in triplicate (N = 5; Two tailed-T test) (**) p<0.01 vs. WT. (B) Confocal microscopy analysis of Lamp-2 surface staining on Ctrl and ASO cells with anti-Lamp2 antibody. Cells are stained with the primary antibody before permeabilization.

The subcellular trafficking of MPR300 is complex and depends on interactions with cytosolic adaptors, such as AP-1, AP-2, GGA 1–3 and the retromer complex [49–51]. Newly synthesized MPR300 can go directly to the plasma membrane where it is quickly internalized to endosomes by an AP-2/clathrin-coated mechanism (the so-called indirect route). However, the majority of MPR300 goes directly from the TGN to endosomes by AP-1 clathrin-coated vesicles (the direct route). From endosomes, MPR300 is retrogradely transported to the TGN, a mechanism that involves the retromer and the AP-1 multi-protein complex. Interestingly, a lack of the AP-1μ subunit (AP1M1) has been previously reported to cause MPR300 mis-trafficking in neurons with a consequent accumulation in the endosomes of μAP-1^-/-^ cells [51, 52]. In order to evaluate whether AP-1 might play a role in the MPR300 and CD defects in our experimental models, we assessed AP-1 expression levels from ASO^*Tg/Tg*^ mice and relative controls. We noted a large decrease in AP-1 levels from ASO^*Tg/Tg*^ mice (Fig 5A), supporting the hypothesis that a reduction in AP-1 prevents MPR300 returning back to the TGN, resulting in its accumulation in endosomes and consequently in an increased exocytosis outside neurons.

It has been recently reported that depletion of VPS35 (an element of the retromer complex involved in the transport of cargo from the endosome to the TGN) [53] might induce α-syn accumulation in lysosomes by increasing the lysosomal turnover of MPR300 [54, 55]. We speculated on the possibility that α-syn overexpression may impair the expression levels of VPS35, as observed for AP-1. We noted that VPS35 expression levels from ASO mice were not significantly different when compared to the corresponding controls (Fig 5A). Moreover, the retrograded transported VPS10p-D receptors Sortilin and SorLA, which both shuttle between the TGN and endosomal system like MPR300, were also not affected in ASO^*Tg/Tg*^. Furthermore, RT-PCR analysis clearly showed that the expression of all these proteins was not altered at the mRNA level (Table 1).

To further test the hypothesis that α-syn overexpression might be related to defects in the AP-1 route, we examined Lamp2 trafficking in Ctrl and ASO cells. Lamp-2 is normally directly sorted from TGN to lysosomes via the endosomes [56], however in the absence of functional AP-1 it would be expected that Lamp-2 is still directed to the lysosomes albeit mainly through the indirect route via the plasma membrane. Technically this would result in an increased Lamp-2 staining on the plasma membrane. In line with this hypothesis, we observed cell surface staining for Lamp-2 in ASO cells but not in Ctrl, further indicating a general trafficking failure in AP-1 sorted proteins (Fig 5B).

### The reduction in MPR300 intracellular levels of ASO cells and neurons is due to increased degradation and secretion to the media

**Fig 6:**
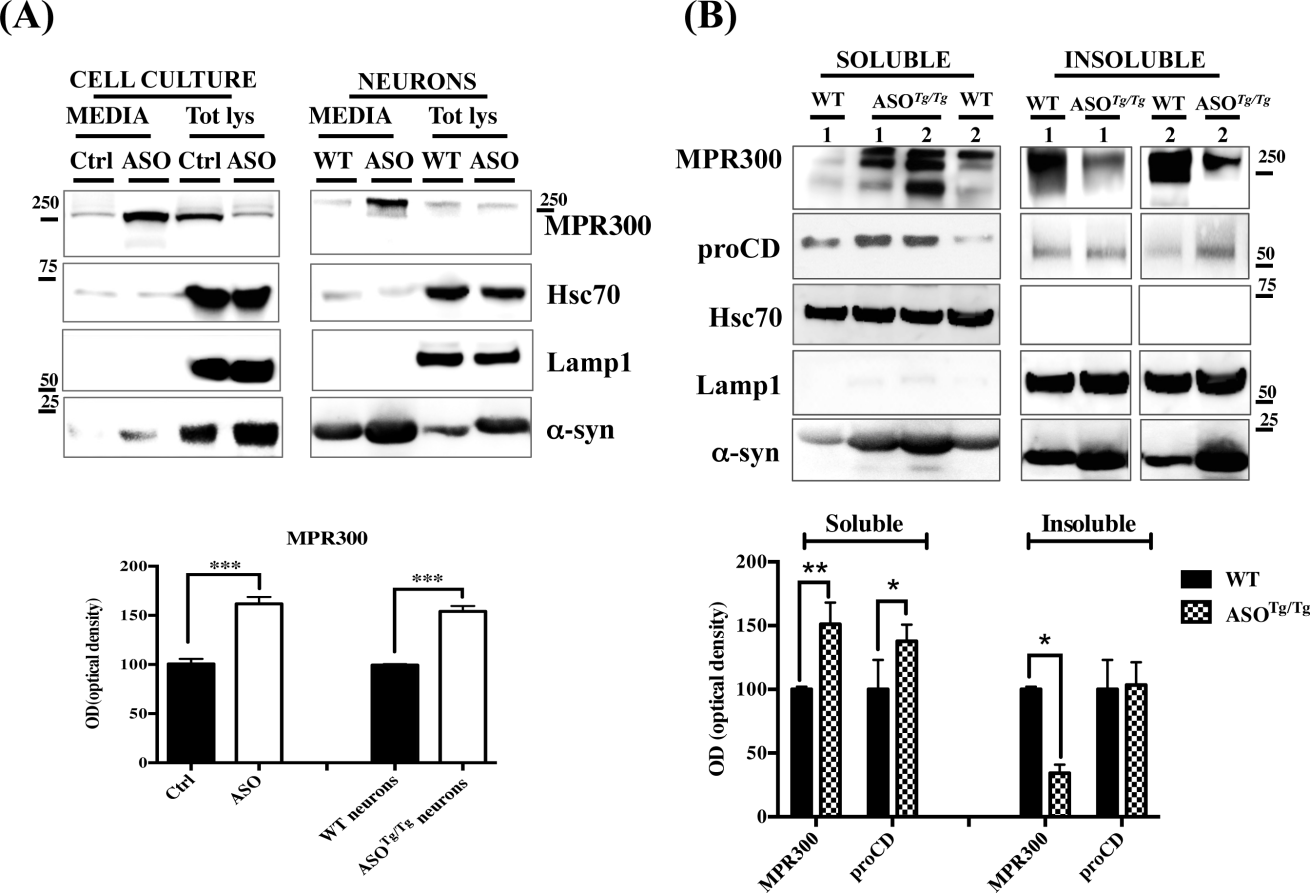
MPR300 protein levels are significantly increased in the soluble fraction of ASO^Tg/Tg^ cortical tissues and in ASO cells. (A) Media collected from ASO cells and the corresponding control (Ctrl) samples and from ASO and WT cortical neurons (after 10 days in culture) were ultra-centrifuged at 100,000 rpm for 12 h. The pellets were solubilized in 2% SDS-loading buffer and (1/10 of the total amount) analysed by WB for MPR300 and proCD. Total lysate was loaded and analysed on the same membranes. proCD was not detectable under our experimental conditions (data not shown). The WB is representative of 3 different experiments. Densitometric analysis of MPR300 levels in media from ASO cells and neurons is reported below. Data were normalized on the basis of Hsc70 levels. The experiments were run 3 times in triplicate (N = 3; Two-tailed-T test) (***) p<0.005 vs. WT. (B) WB analysis for MPR300 and proCD were performed in the soluble and insoluble fractions from cortical tissues of ASO^*Tg/Tg*^ mice and the correspondent WT samples. Hsc70 (soluble and/or secreted marker protein) [63, 64] (protocol is described in Methods) and Lamp1 blot were used as respective positive and negative controls of the soluble and insoluble fractionations. The lack in Lamp1 blot signals in the media of cells and neuronal cultures indicates that there was no cell leakage under our experimental conditions. Optical density analysis is reported below. Data were normalized on the basis of Hsc70 (soluble) or Lamp1 (insoluble) levels and expressed as % of WT. The experiments were performed 4 times in triplicate (N = 4; Two tailed-T test) (*) p<0.05 and (**) p<0.01 vs. WT.

Under normal conditions, CD is efficiently sorted to the endocytic system with very little being secreted from the cell [40]. However in some pathological conditions, proCD and CD can be secreted into the extracellular space where these enzymes have been shown to contribute to the invasive and metastatic properties of cancer cells [40, 57]. A similar fate has also been described for MPR300 [58, 59]. To address the hypothesis that the reduction in MPR300 intracellular levels also might be due to its secretion we first examined MPR300 levels in media from ASO cell culture and neurons and in the correspondent controls. We found a significant increase in MPR300 and α-syn secreted levels both in media from ASO cell and neurons (Fig 6A), supporting the hypothesis that α-syn overexpression affects the endo-lysosomal pathway resulting the mis-sorting and consequent secretion of MPR300. Notably, MPR300 levels were not different between WT and ASO neurons (Fig 6A), suggesting that neuronal cultures might require longer time in culture before showing significant evidence of reduced MPR300 expression levels. Furthermore, the finding that secreted MPR300 levels are significantly increased in the media from ASO neurons after 10 days in culture suggests that MPR300 secretion might precede its decrease that occurs later in neuronal cultures. Interestingly, proCD was undetectable in media both of cell culture and neurons, suggesting that it is secreted in extremely low amount in our experimental models (data not shown).

Additionally, we analysed protein levels of proCD and MPR300 both in the soluble (mostly containing cytosolic proteins) and insoluble (mostly containing membranes) brain tissue fractions of ASO^*Tg/Tg*^ mice and relevant controls. Notably, we found a large increase in soluble MPR300 in ASO^*Tg/Tg*^ mouse brains that was consistent with its corresponding decrease in the insoluble fractions (Fig 6B). ProCD was also higher in the soluble fraction of ASO^*Tg/Tg*^ mice when compared to WT. However, due to the low expression levels of proCD in the correspondent insoluble fraction, we detected no significant changes (Fig 6B).

**Fig 7:**
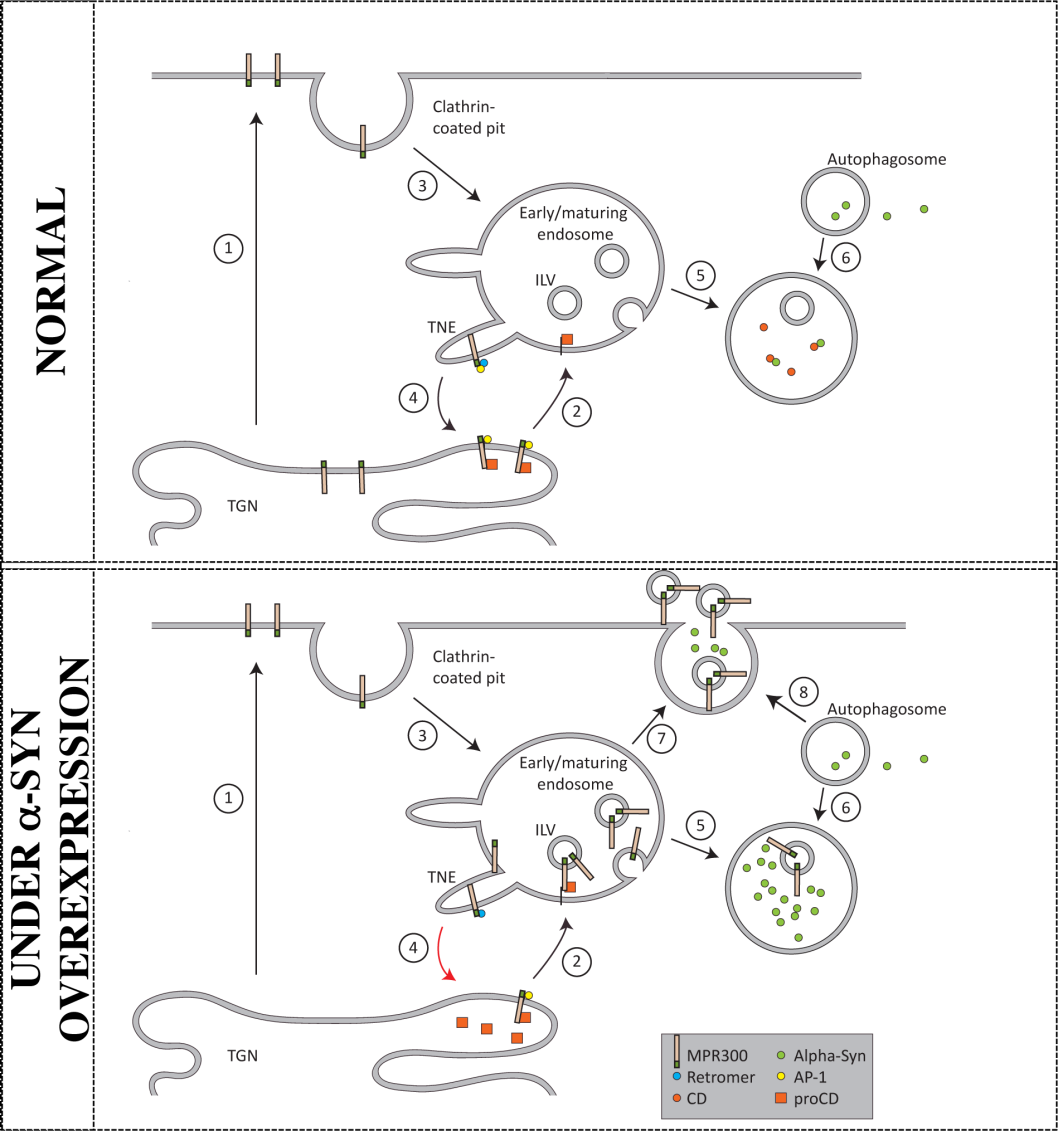
Schematic drawing of MPR300 trafficking in cells. The top panel displays how MPR300 traffics in neurons under normal physiological conditions. In short: after final glycosylation in the Golgi, MPR300 can either be secreted by a default route to the cell membrane (1) or actively transported to the endo-lysosomal system by interacting with AP-1 and GGA 1–3 (2). MPR300 on the membrane can be internalised via a coated pit to reach the endosomes (3). When MPR300 reaches endosomes, the retromer complex and AP-1 facilitates the retrograde transport to the TGN (4). More than 90% of endogenously expressed MPR300 is estimated to enter the TGN-endosome shuttle at any given time and this transport is essential for the transport of CD to lysosomes (5). α-Syn enters the lysosomes via autophagosomes (6). The lower panel reports how a reduced MPR300 retrograde transport in neurons likely influences proCD sorting in PD neurons (4). The reduction of MPR300 reduces the transport of proCD to the endosomal system and its processing into the mature form of CD. Moreover, MPR300 accumulates in the membrane of late endosomes and in the intraluminal vesicles (ILV). Finally, the only escape from endosomes is by lysosomal degradation or secretion (in this latter case the ILV are termed exosomes).

In conclusion, we hypothesize that whereas MPR300 under normal conditions shuttles between TGN and late endosomes, overexpression of α-syn results in a reduction of AP-1 that consequently compromises the retrograde transport (Fig 7, route 2 and 4). A defect in MPR300 transport from endosomes to TGN will cause an accumulation of MPR300 in late endosomes. This accumulation will eventually trigger MPR300 to follow the default pathways out of endosomes, which is to the lysosomes or to be secreted. Inefficient MPR300 trafficking will then be followed by reduced proCD transport to lysosomes. Deficiency in retrograde MPR300 transport can also be caused by a defect in the retromer complex as observed in the case with familiar mutations in the VPS35 gene and by other cytosolic adaptor proteins [60, 61]. Although the VPS35 expression was not affected in our PD models, we cannot exclude that other proteins involved in the retrograde MPR300 transport might be disturbed and consequently influence MPR expression and trafficking in PD neurons.

### MPR300 is reduced in human PD samples

**Fig 8:**
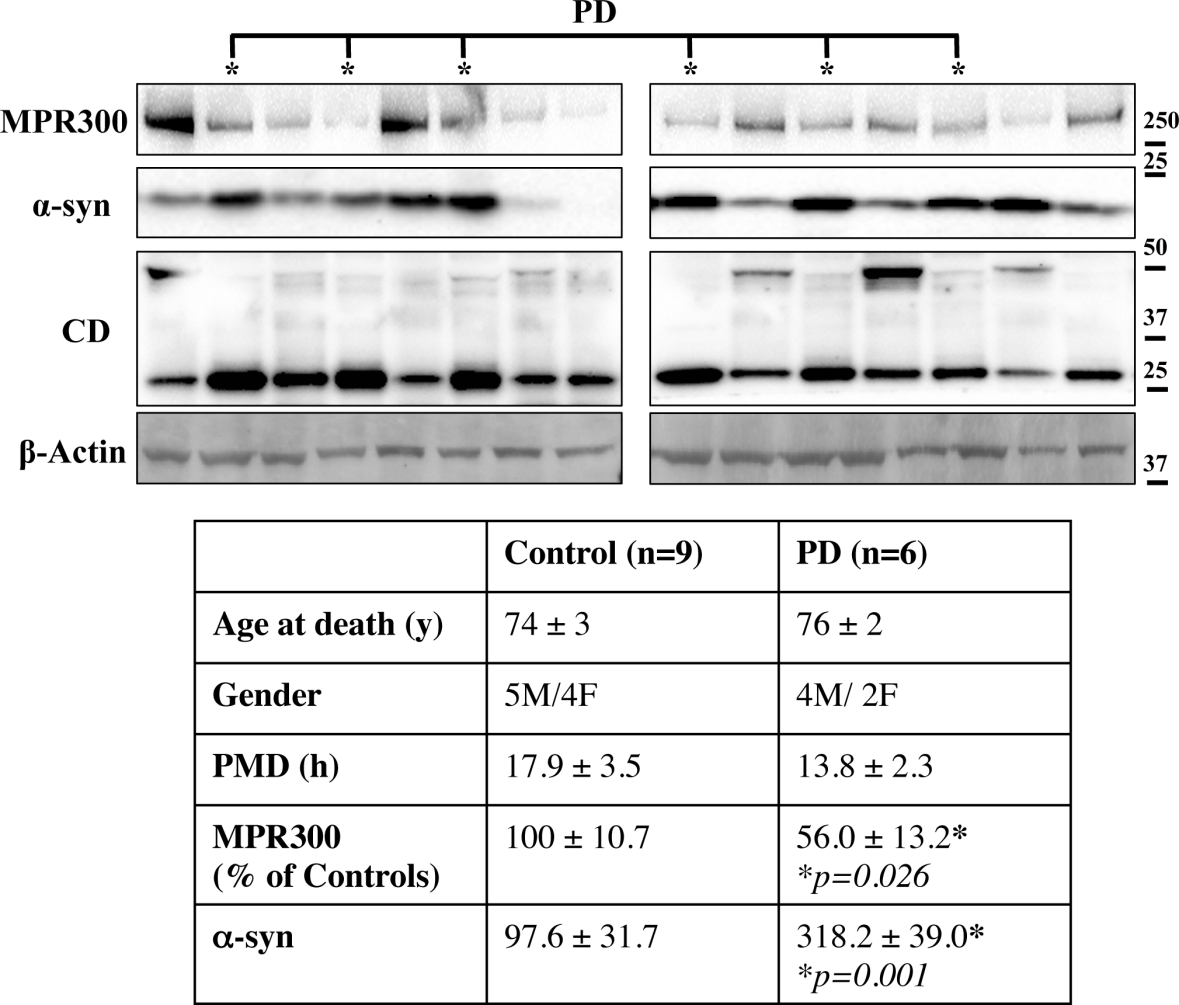
MPR300 is decreased in human brain from patients with early PD. WB of MPR300 and α-syn in SDS-soluble protein lysates generated from the anterior cingulate cortex of 6 autopsy-confirmed patients with Braak Lewy body stage 4 (PD) [65] versus 9 controls as detailed in the table below reporting their demographic details. Immunoblot data was normalized to β-actin and a multivariate analysis performed to determine if MPR300 or α-syn were changed in PD. Age and post-mortem delay were included as covariates. The results show a significant reduction in MPR300 protein and an expected significant increase in α-syn levels in PD anterior cingulate cortex. Values are given as mean ± SEM and * = p<0.05.

Finally we investigated whether MPR300 expression was reduced in PD patients early during the progression of the disease. To address this we performed WB for MPR300 and CD in SDS-soluble lysates generated from PD patients selected with an early Braak Lewy body stage of 4 in which α-syn deposition is largely confined to brainstem and basal forebrain regions. The same cases have previously been used to measure CD levels with the finding that the protein was increased in PD [22]. While this case selection process limited the number of PD cases for evaluation, this cohort provided an opportunity to better understand earlier pathogenic events compared to late end-stage PD cases. In these cases and matched controls, tissue from the anterior cingulate cortex were evaluated where we have previously shown early biochemical changes in the presence of increased SDS-soluble α-syn, but not yet Lewy body pathology [22]. WB data were normalised to β-actin and multivariate analysis (covarying for age and post-mortem delay) used to determine changes in α-syn and MPR300 in the PD group compared to control. The analysis revealed a significant decrease in MPR300 protein in the PD group (Fig 8) and as expected α-syn was significantly increased (Fig 8). Age (p = 0.21) and post mortem delay (p = 0.10) had no significant influence on the protein levels of MPR300 or α-syn.

Interestingly, CD appeared to be almost completely processed to generate the active mature form (CD 32Da) in the postmortem tissues from PD patients. Based on the present results and the results from data in a primate PD model [62], we predict that any increase in CD would be mis-localised at this early disease stage. Thus, defects in MPR300 may precede the mis-localisation of CD resulting in the increase of insoluble α-syn and ultimately a decrease in mature CD in appropriate functional compartments as Lewy pathology develops. These results are supported by other tissue studies in the very vulnerable PD substantia nigra showing reduced CD in association with Lewy pathology [5]. Our results are consistent with such pathology occurring following defective MPR300 transport.

## Conclusions

We present here a new scenario for PD in which α-syn overexpression induces defects in AP-1 mediated MPR300 trafficking and sorting using, cell lines, mouse models and clinical samples. Accordingly, a decrease in MPR300 levels, and its altered shuttling between endosomes and the TGN, causes an improper cathepsin D (CD) trafficking to lysosomes and likely α-syn accumulation in neurons, finally related to PD disease. These results pinpoint MPR300 as a potential biomarker for PD prediction.

## Abbreviations

TGN: Trans Golgi-network
PD: Parkinson's disease
ALP: autophagy-lysosomal pathway
VPS35: vacuolar sorting protein 35
CD: cathepsin D
MPR300: 300 kDa mannose-6-phosphate receptor
SNCA: α-syn gene
